# Inaugural Address of the President of the Medical Society of the State of New York, Dr. Thomas F. Rochester, Delivered in Albany, June 20th, 1876

**Published:** 1876-06

**Authors:** Thomas F. Rochester

**Affiliations:** The President; Buffalo, Erie Co., N. Y.


BUFFALO

Medical and Surgical Journal.

VOL XV. JUNE, 1876. No. 11.

### Original Communications.

:o:

ART. I. *Inaugural Address Delivered at Albany, June* 20, 1876, *at the opening of the Seventieth Session of the Medical Society of the State of New York.* By the President, Thomas F. Rochester, M. D., of Buffalo, Erie Co., N. Y.

Fellow Members and Delegates :

We meet again after an interval of nearly seventeen months—it is hoped to the satisfaction and increased convenience of all, at the change in time of our annual assembling. This has been accomplished somewhat informally, and not quite in the manner contemplated by the Society. At the meeting in February, 1875, when the change was resolved upon, a committee was appointed to secure the necessary legislation. The committee took the requisite steps, and so reported while the Society was still in session, but the action thus initiated, appears not to have been carried to its legitimate conclusion, probably through the omission to act, on the part of the legislators to whom the bill was entrusted. In this dilemma, it was thought by the older members of the Society, resident in Albany, that the desired end could be accomplished by holding an informal meeting at the usual time, and adjourning to the third Tuesday in June, coupled with a resolution, that the annual meetings, should thereafter be held at that date. For a more complete account of this action, you are referred to the minutes of the Secretary. It is hoped that the conclusion, that legislative action was unnecessary, was correct, but if there is any doubt upon the subject, that action may yet and should be taken.

It is customary for your presiding officer to refer to the general sanitary condition. It is with sincere thankfulness that the speaker is able to state, that no alarming epidemic and no unusual mortality has been noted. In certain localities scarlet fever and diphtheria have been prevalent, but have not extended over a large portion of the state. During the spring months of 1876 a catarrhal influenza of unusual duration and severity has prevailed, extending from the seaboard to the great inland lakes, and in fact over a large portion of the North American continent, resembling more closely the noted equine epidemic of a few years since, than that which usually attacks the human family. It was attended with extreme prostration, was tenacious in its hold, was but little influenced by quinine, antiseptics or opiates, and very distinctly required the free use of alcoholic stimulants and nutrients. Where it attacked the aged or those enfeebled by chronic disease, it often developed fatal lesions. In infants and quite young children it was in many instances attended with convulsions or prolonged nervous tremors. Not many years since an influenza epidemic was regarded as a fore-runner of cholera. It is most earnestly to be hoped that this belief will not be verified in this centennial year of 1876. Just preceding the outbreak and during the prevalence of this disorder, the barometer was unusually low, and it was notably observed, that the fluctuations of the disease corresponded with those of the mercurial tube. Thus affording an excellent illustration of the importance of statistical weather reports, and of the relation existing between atmospheric changes and sanitarian vital conditions. It has long been the desire of Gen. A. J. Myer to embrace observations pertaining to health and disease in the bureau of which he is the eminent head, and there is no doubt that they would be quite as valuable to the medical profession, as those now considered indispensable by navigators and agriculturists. Every effort should be made by scientists to accomplish this object. We of Buffalo generally speak of him as Dr. Myer. We are proud to claim him as an alumnus of our Medical College. He was prominent as a surgeon in the army, when he invented the code of signals, which led to the formation of the Signal Corps, at the head of which he was necessarily placed, with a military position and title which transferred him from the medical department. As an evidence of his turn of mind and great ingenuity, it may here be mentioned, that when a student he formed an alphabet for the deaf and dumb, which has been adopted in many public institutions. By all means, let us do all that lies in our power to bring him back to his legitimate professional sphere, by encouraging the idea of a health with the weather bureau. Of the thereapeutical measures that now occupy the medical mind, decidedly the most prominent is that known as the apyretic treatment of disease. It is a subject demanding the gravest consideration. Advocated and practiced by prominent European practitioners, for the last three years, and nearly as long by a few of our own, it has within the last eighteen months gained a strong foothold in our midst, principally through the instrumentality of the translation of Ziemssen's Cyclopedia of the Practice of Medicine, put forth in the most attractive form, by the well known medical publishers, William Wood & Co., of New York. It is based upon the idea of the destructive effect of elevation of temperature upon nerve and muscle tissue, and certainly presents an immense number of clinical observations and data, sustaining the theory of the necrobiotic metamorphoses induced thereby, and also apparently demonstrating the wonderfully beneficial effect of various agents administered and applied solely with reference to their power in abating heat. First and foremost of these is water, in warm, cold and ice-cold baths—ice to the spine, ice to the abdomen and other parts of the surface—in short, water applied with a persistence and repetition such as Priessnitz never even dared to think of ; next quinine at very short intervals, counted by minutes, in doses rivaling those of the malarious southwest, where the measure is not by grains, but by the teaspoonful ; next arterial sedatives such as digitalis, aconite, veratrum, viride and gelseminum, and perhaps other equally powerful and often dangerous drugs. Is it not best to be cautious about embracing too enthusiastically this new *therapeia?* Let us not be misunderstood—the dangers of extreme and continued pyrexia, in expressive Boston parlance—fever waste, are fully appreciated, and they should be averted by all reasonable measures, but it is respectfully suggested that in giving our whole attention to a product of disease, we may overlook or lose sight altogether of morbific conditions not less important and formidable. Let us give all credit to the medical savans of Germany—none have surpassed, few have equalled them in any department—but their enthusiasm may sometimes bias their reason. They have had the misfortune to produce a Hahnemann, with his infinitesimals—they may possibly err in the opposite direction, with their maximals. They make of quinine a panacea. It is stimulant, it is sedative, it is tonic, it is alterative, it is anti-phlogistic it is astrignent and constringent, it is apyretic, it is in fact of universal powers, properties and application. It is *totis prescribenda*. There is danger that this inestimable article of materia medica will be brought into discredit, from its indiscriminate and excessive use, and that while from the same causes, its price will be so enhanced as to put it beyond the reach of the poor (those who need it most), the temptation to adulterate it, will cause many dealers, to dispose of a very inferior article. During the late war, immense quantities were supplied to the army, in large part consisting of cotton fibre.

Among the many diseases for which quinine in full doses is now advantageously prescribed is inflammatory croup. This is particularly specified, to put on record as a matter of historic justice, the fact, that Dr. H. N. Eastman, of Owego, N. Y., and Dr. D. McFarland, of Williamsburg, L. I., each independently of the other, advised the same nearly twenty-five years since, in articles published in the *New York Journal of Medicine*. It will be remembered that a "Note on Salicylic Acid" was the title of a very interesting paper at our last meeting. It was treated from a purely chemical and scientific stand-point, and it must be a source of great gratification to its distinguished author—Prof. E. R. Squibb—to find that the deductions drawn by him have been more than realized in practical use. As an antiseptic it is probably not inferior to carbolic acid, while it is free from its objectionable taste and smell, and is much more suitable for internal administration. As an apyretic it has developed remarkable qualities. By many it is regarded as a specific in acute rheumatism. In this disease, to the writer, its action and effect has appeared to be similar to that of propylamine. A new antiseptic, the sulpho-carbolate of soda, is now sub-judice. It is extravagantly lauded by several writers in recent British periodicals for its control of measles and scarlet fever. Among many meritorious recent publications, special attention is called to the carefully prepared statistical paper of Prof. E. L. Keyes, M. D., of New York, on "The Effect of Small Doses of Mercury in Modifying the Number of the Red Blood Corpuscles in Syphilis."* It appears to demonstrate that under its judicious use, either alone or combined with iodide of potash, that these are increased in number. A fact, if it prove to be such, at variance with the general opinion—and of the utmost importance, in relation to the saddest scourge with which the human race is afflicted, as the penalty of vicious indulgence.

Mention should also be made of the essay of Alex. Hutchins, M. D., of Brooklyn, N. Y., on the "Nitrite of Amyl," being his inaugural address as President of the Medical Society of the County of Kings. It is a full and thorough examination of the physiological and therapeutic action of this agent, and certainly offers good reasons for the careful employment of it, in many forms of severe and intractable spasmodic disease. It is so prompt, powerful and peculiar in its effects, that although its properties have been known for several years, its use has been limited, perhaps, from a wholesome timidity. Should it prove to be as safe as it is claimed to be effectual, it is a great boon to suffering humanity. There is much concurrent testimony respecting its instantaneous relief of angina pectoris. With respect to this, however, and to all active "remedies" of whatever class—the needed lesson of the day, as agreed by all thoughtful and experienced practitioners, is *caution*. We are fond of saying that much less medicine is given

*Reprint from Am. Jour. Med. Sciences for January, 1875.

now than formerly, and this is undoubtedly true as respects quantity, but there are some other considerations. Through the wonderful advances in physiology and pathology, and in accurate diagnosis and prognosis, medicine in these matters has become almost an exact science. Chemistry, too, has become so precise that from form it predicts properties, and penetrating into the hidden depths of nature, gives us the vital essence of material under the name of active principles. Pharmacy, the handmaiden of chemistry, prepares these alkaloids, these acids, these extracts, these active principles, with practised and dexterous hands, so that neither the eye, or the tongue or the nose is offended, and the therapeutist, eager and ambitious, with these at his command, and made wonderfully rapid, and energized through hypodermic application, seeks for an immediate and specific effect. He is as anxious to be as brilliant and as sure in his medication, as he is in his diagnosis—and here lies the danger—ardently as it is to be desired, it is feared that the time is far distant when that branch of our profession which embraces the science of materia medica can be called exact. While it is to be hoped that many specifics may be added to the few now known, there is also another side in the treatment of disease, and the physician is wise who thus questions himself—Does this patient require medicine? If so, how little will suffice? Not how much will he tolerate? Is not the last query propounded too often, does it not occasion many errors and mistakes?

In surgery we are not aware that there is anything of special moment to chronicle, but we regret to state that the bandage of Esmarch, from which so much of positive good has been derived, is not always innocuous—its use having been followed in several instances by fatal embolism of the pulmonary and other arteries.

Your attention is now invited to the question of the preliminary education of medical students. It is again brought to your notice by the special request of the Erie County Medical Society, at its fifty-fifth annual meeting in January, 1876. It will be remembered that in February, 1872, the Committee on Medical Education, of the Medical Society of the State of New York, endorsed the action of the American Medical Association in 1871—and that among other resolutions embodied in their report, which was adopted, was the following: *Resolved*, "That no member of any medical organization represented in this society, be permitted to receive a student into his office until such student presents the certificate of a Board of Censors, showing his qualifications to enter upon the study of medicine." It is claimed that this is not observed, that it is ignored both by County Societes and by individual practitioners—that the Medical Societies of Erie county, and Ulster county, and the Lake Erie Medical Society, (an association formed from Chautauqua, Cattaraugus and Wyoming counties) are the only bodies who have tried to carry it into effect· Of these, the Erie County Medical Society is the pioneer, having at its semi-annual meeting in June, 1872, bound itself to conform to the requirements of the State Society—and since January, 1873, has, through its censors, worked diligently in this direction, although as they claim, with but partial success. By all means, let the State Report of 1872 be re-affirmed, and may each member of this society on his return home, make it his business to see that his County Society does its duty. Some censure has been passed by those whose zeal has outrun their discretion upon Medical Colleges in this matter. Some of the medical schools require a preliminary examination of every student proposing to matriculate, and all should do so. The examining board of course being selected from the respective faculties. Unless this is held as equivalent to an examination by a board of County Society Censors, discrimination against students from New York, in favor of students from the other States and from Canada, is unavoidable. Harvard, always wise and progressive, has shown us how to solve this difficulty by establishing a three years graded course of instruction, the school thus of necessity becoming the preceptor, and being responsible intoto for the pupil. Thus no loop hole is left by which, between school and preceptor, improper students may manage to glide into a degree. This, by no means, is meant to do away with the great advantage of having a private preceptor. It is hoped that no one will object to spending a few moments upon a subject, which, from its pre-eminent importance, can never become hackneyed. We refer to Hygiene. The State authorities are at length aroused, and have instituted a series of sanitary measures. Among others a State Topographical Survey. The director of this survey, Prof. J. F. Gardner, has been invited to meet us at this meeting through the instrumentality of that earnest sanitarian, Dr. Elisha Harris, and will make some remarks pertaining to malarial areas. It is unnecessary to say that in this important work he will have the co-operation of the whole profession. The avenues of Hygiene are so many, and so broad, (and yet many of them so narrow, that they have to be searched out), that there is no loss for subjects. It is proposed at this time to bring only one point of the field before you, and that is, School Hygiene. This has interested the writer for many years, and you will pardon him if he presents his ideas, by reading to you some extracts from a public lecture delivered by him in Buffalo in February, 1869. "We are proud of our public schools; education is free to all, but it is not in every instance the unmixed blessing it seems. It is acquired at too great an individual risk.

"On the proudest avenue of this city is a three storied brick building. The room is heated with coal stoves, the ceiling is low, the light is but moderate, and there is no provision for ventilation. The seats are short, narrow and close together. Eighteen months ago a meeting of the residents of the district was convened, to consider the propriety of repairing the old, or of building a new structure. The Principal of the school, in reply to inquries, stated that the room was always full; that three children had to sit where there was only room for two; that they were packed so tightly that it would be impossible for the children all to rise upon their feet at once; that there was no place to hang up their outer garments, even if they were wet, and that when school was dismissed, if a boy should drop his cap, he could not stop to pick it up, so great was the rush and the crush. The tax payers, a majority of them, buttoned up their pockets and voted to repair, one of them, a wealthy professional man remarking "that it was a much better school house than the one he had studied in." Not so, sir. The little country wooden structure, with its rattling windows, its gaping cracks and its broad-mouthed fire, place was infinitely superior.

"On the 9th of February, 1869, the School Committee of the Common Council, with the Superintendent of schools, made a tour of inspection. I make a few extracts from the report of the same: No. 7. "The primary department was found to be running over with little children, who had hardly room to breathe and stretch out their little arms." No. 11. "It is a perfect hive of children." No. 31. "The Primary Department has three hundred and forty scholars, but was calculated only to hold one hundred and eighty. They sit everywhere." No. 15. "The Primary Department contained three hundred and twenty scholars yesterday." From eight to twelve hundred feet of cubic air, is the amount of space that is required to be allotted to each individual in the U. S. Military Hospitals. In British India each jail prisoner has by legal enactment, six hundred and forty-eight cubic feet of air. In public school No. 15, each poor child has but fifty-six cubic feet of air. All the public schools are not like these, but that there should be one such is a burning shame. It is the *Primary Department.* This place where the child of five years old and upwards, the little darling, that yet needs a mother's watchful eye and care, is subjected to such infamous trial and exposure. No wonder that scarlet fever, diphtheria, typhoid fever, and blood poisons of every description, are more or less prevalent. A large proportion of these dread disorders are generated in and propagated by our public schools, and the sordid man who votes against a tax for health and education, cannot guard his own spacious and luxurious abode against the malady his own avarice has helped to originate. But acute diseases are not the only results of this criminal crowding. Tuberculosis, scrofulous and brain affections, developed at various periods, may be traced, but too often, to the same source. Better for society, and better for themselves would it be, that these infants were not educated at all than at such risk." The counterpart of this picture is to be found in every large city in our land. What is the remedy? No child under ten years of age should be sent to a public school, and every school district should have a competent and well paid medical director, who should devote himself thoroughly and conscientiously to the many hygienic duties of the position. It would not involve an increased expense; on the contrary, it would be to the community a most wise and economical procedure. School Hygiene is often referred to in medical und secular journals, but so far, with little practical result. In this connection, the paper of Dr. David Webster, of New York, read at a late meeting of the American Social Science Association, is most apposite, demonstrating by very extended clinical examinations in various cities, the immense injury done to the visual apparatus by the prevailing surroundings and methods of school study. My brethren, cannot we, as representing the profession of the State, take some action which shall inaugurate and perpetuate a reform? If not, we cannot be held guiltless of this annual "slaughter of the innocents."

This society has now become so old and so large, that a sad duty necessarily always devolves upon its presiding officer—the mention of those of its members and delegates dead within the year. Of the former, Professor Charles B. Coventry, of Utica, who adorned our presidential chair in 1855, passed away at the ripe age of 74 years, on Feb. 23d, 1875, honored and distinguished in his professsion; of gentle and winning manners, and especially noted for his kindly bearing toward his younger brethren.

On Dec. 3d, 1875, Prof. J. H. Armsby, and on Dec. 19th, 1875, Peter McNaughton, both old and esteemed residents of Albany, both highly eminent in their profession, and for a quarter of a century active and prominent members of this association.

In New York, in December, 1875, very suddenly, Dr. John R. Van Kleek, a member of the board of trustees of the Physicians, Medical Aid Society, and much esteemed by his associates in every relation.

In Lansingburg, Jan. 19th, 1876, George H. Hubbard, earnest and zealous, and a great loss to the community and to this society.

In Auburn, March, 1876, James W. Wilkie, of most excellent repute, and held in great affection by his patients.

All of the above were permanent members. Of those who have served as delegates, the first on the list is Edward Delafield, of New York, one of the most eminent physicians that great city has ever boasted. He died on the 13th of February, 1875, aged 81 years. His funeral was solemnized at Trinity church, and, sad and unwonted spectacle, his two brothers (septenarians) were his mortuary companions, all three, by a singular fortuity having died almost simultaneously.

Foster Swift, of New York, in his 42d year, with a manly heart, breathed out his gentle soul at Santa Cruz, May 10th, 1875. Son of Gen. Joseph Gardiner Swift, the first graduate of West Point, and grandson of Dr. Foster Swift, a Surgeon in the Revolutionary army, he could not help but be patriotic. He accompanied the 8th N. Y. Regiment to the late war at the first call to arms, as Surgeon, and his cool reply when summoned to surrender, at the disastrous battle of Bull Run, "Yes! but wait until I tie this artery!" rang through the land.

Those of us who were present at our last meeting, remember the tall figure and dignified bearing of Ernest Krackowizer. He died at Sing Sing, Sept. 22d, 1875. He was one of those European scholars, who burnish the art escutcheon of the New with the lustre of the Old World.

F. W. Root, of Hamilton, Madison Co., N. Y., died May 8th, 1876, a modest man, and an excellent practitioner. There may be others, but this as far as is known to the writer, closes the list. Alas! too long. The Committee on Necrology will have a painful but most honorable task, in writing the memorials of so many most excellent men. It has been suggested to the speaker by one of the ex-Presidents of the Society, that our influence and usefulness might be extended by an additional number of Vice-Presidents. It would certainly enable us to do honor to some of our associates, and the matter is submitted to you for consideration. The Committee of Publication deserve our thanks for the promptness with which they issued the last volume of Transactions, and also for the great improvement in its style and appearance, over that of its predecessors. It is submitted that an alphabetical list of permanent members, a directory in fact, would be more convenient than the present arrangement; it should appear in each volume. The official acts of the speaker have been very few.

On the 8th of February, 1875, he was consulted by the Secretary as to the M. H. Cash prize, the amount in the treasury to that fund being but \$50, while latterly, from accrued interests, \$100

had been paid. He advised that the interest of the fund, yearly and accrued, alone could be paid, there being no authority to make up the $100, from the general funds of the society. If there is any question on this point, it should be settled that contestants may know, for just how much money, as well as honor they are competing.

On the 9th of June, 1875, he signed the State degree of Doctor of Medicine for Hermann Mynter, M. D., a native of Denmark, and graduate of the Medical School at Copenhagan, on the written certificate and recommendation of C. C. Wycoff, M. D., Harvey Jewett, M. D., and Caleb Green, M. D., State Censors, before whom Dr. Mynter had passed the requisite examination.

On the 23d of March, 1876, he received a communication from the President of the Kings Co. Medical Society, requesting him to write to the Senator of the 31st District, respecting a bill before the Judiciary Committee, "to protect physicians in suits for malpractice." Senator S. S. Rogers responded March 28th, 1876. "The bill of which you write, was reported unfavorably some time ago." The writer is informed by competent authority that no bill requiring bail for the costs of the suit, can be passed, it being impracticable.

On the first day of January, 1876, he received through the Secretary the subjoined communication:

INTERNATIONAL MEDICAL CONGRESS.
PHILADELPHIA, 1876.
All communications should be addressed to the Corresponding Secretary.

PHILADELPHIA, Dec. 13th, 1875.

*To the President and Members of the N. Y. State Medical Society.*

GENTLEMEN:

It is proposed that an International Medical Congress be held in this city, in September, 1876,—advantage being taken of the International Exposition celebrating the One Hundredth Year of our national existence, as a favorable occasion for assembling our professional brethren from all parts of the civilized world.

The main object of the meeting will be the discussion of topics belonging to the various branches of medical science.

The preliminary arrangements for holding the Congress have been entrusted to a commission composed of representatives from the different societies in this city. What this body has already done, will appear from the accompanying circular.

On behalf of the Commission, we respectfully request that you will appoint delegates to represent you in the International Medical Congress in 1876.

It will facilitate the business of this Committee, if, upon the appointment of such delegates, you will cause to be transmitted to us a list of their names and addresses.

We have the honor to be, gentlemen, with much respect,

Your obedient servants,

L. D. Gross, *President.*

D. G. Brenton, *Corresponding Secretary.*

Note.—The proposed plan of organization entitles each State or Territorial Medical Society to the same number of delegates to the Congress, as the State or Territory has representatives in the Congress of the United States.

To this he responded by sending to Dr. Gross the following names:

Delegates from the Medical Society of the State of New York, to the International Medical Congress to be convened at Philadelphia, Monday, September 4th, 1876:

ALONZO CLARK..............23 East 21st Street, New York.
FRANK H. HAMILTON.........43 West 32d Street, New York.
B. FORDYCE BARKER.............85 Madion Avenue, N. Y.
EDWARD H. PARKER....... ..........Poughkeepsie, N. Y.
THOMAS HUN..............................Albany, N. Y.
FREDERICK HYDE........ ...............Cortland, N. Y.
HENRY W. DEAN.......... ............ .Rochester, N. Y.
JOHN P. GRAY..................................Utica, N. Y.
L. I. TEFT.................................. Syracuse, N. Y.
J. V. P. QUACKENBUSH......................Albany, N. Y.
JAMES P. WHITE.......... ........... ....Buffalo, N. Y.
GEORGE BURR.........................Binghamton, N. Y.
S. O. VANDERPOEL ....... ............Quarantine, N. Y.
G. A. DAYTON..................Mexico, Oswego Co., N. Y.
WM. C. WEY,......................... .. ...Elmira, N. Y.
C. R. AGNEW......................19 East 39th Street, N. Y.
B. F. SHERMAN...........................Ogdensburg, N. Y.
E. M. MOORE....... .....................Rochester, N. Y.
FRANCIS BURDICK.............Johnstown, Fulton Co., N. Y.
GEO. J. FISHER........ ....................Sing Sing, N. Y.
HARVEY JEWETT...... ..............Canandaigua, N. Y.
ELLSWORTH ELIOT.............48 West 36th Street, N. Y.
JULIUS F. MINER...... ...... ...............Buffalo, N. Y.
W. H. BAILEY.................................Albany, N. Y,
E. R. HUN.................... ..............Albany, N. Y.
C. H. PORTER.... .. .......................Albany, N. Y.
J. FOSTER JENKINS........................Yonkers, N. Y.
A. M. VEDDER............................Schnectady, N. Y.
H. D. DIDAMA..............................Syracuse, N. Y.
E. R. SQUIBB............................Brooklyn, N. Y.
ALEXANDER HUTCHINS.... .............Brooklyn, N. Y.
H. D. VOSBURG.... .........................Lyons, N. Y,
AUSTIN FLINT, Jr.............14 West 33d Street, New York.
JOHN D. BUTTON..........................Auburn, N. Y.
THOMAS M. FLANDREAU...................Rome, N. Y.

All have consented to serve except Dr. Alonzo Clark, who was obliged to decline, and Dr. Thomas Hun, who is absent in Europe. Dr. J. C. Hutchinson, of Brooklyn, was nominated in place of Dr. Hun. The vacancy left by Dr. Clark has not been filled. Dr. Frank H. Hamilton has been designated as Chairman, Dr. Clark having been previously tendered the position. It is unnecessary to state of how great interest and importance this International Medical Congress must be. It is hoped that all the delegates from the State Medical Society will be able to attend, and while the speaker would not presume to influence in any way their actions, he trusts that it will among other advances, favor a uniform system of weights, measures and symbols, throughout the medical world.

It remains, gentlemen, for the speaker to express to you his cordial and heart-felt thanks for the high honor you have conferred upon him, in selecting him to preside over your deliberations. Totally unversed in parliamentary usages, he begs for your kindly consideration and assistance in the discharge of the duties belonging to his position. The Society is now organized and ready for the transaction of business.

